# The effects of acute physical and cognitive exercises on sequential motor skill learning: An exploratory study

**DOI:** 10.1371/journal.pone.0327725

**Published:** 2025-07-11

**Authors:** Guillaume Digonet, Thomas Lapole, Gabrielle Pouilloux, Ursula Debarnot

**Affiliations:** 1 Universite Claude Bernard Lyon 1 (UCBL1), Laboratoire Interuniversitaire de Biologie de la Motricité, Villeurbanne, France; 2 Universite Jean Monnet Saint-Etienne, Lyon 1, Université Savoie Mont-Blanc, Laboratoire Interuniversitaire de Biologie de la Motricité, Saint-Etienne, France; 3 Institut Universitaire de France, Paris, France; University of Utah, UNITED STATES OF AMERICA

## Abstract

Physical or cognitive exercises before motor skill learning are increasingly examined as a means to optimize performance during acquisition and consolidation processes. However, their respective effects remain underexplored in explicit sequential motor learning (SML). In this study, we examined whether different types of acute exercises such as sprint interval exercise, cognitive exercise, and a combination of both executed prior to explicit SML could modulate motor performance during acquisition and consolidation relative to a control group performing a neutral task. A total of 60 participants were randomly assigned to one of the four experimental groups. The psychophysiological modulations induced by the exercises were assessed using the NASA-TLX questionnaire and blood lactate measurements. Motor performance was evaluated at the beginning and the end of SML acquisition (early- and late-acquisition) and following delayed consolidation at 24h and one week later. Physical exercise elicited an increase in both lactate levels and subjective physical demand, while cognitive exercise increased mental demand. Overall, motor performance improved during both acquisition and consolidation at 24h and a week later, but without any difference between groups. Our findings suggest that neither sprint interval exercise, cognitive exercise, nor their combined execution prior to explicit SML significantly influences motor skill performance during acquisition and consolidation compared to a control intervention, although this absence of significant effects should be interpreted with caution.

## 1. Introduction

Sequential motor learning (SML) involves the integration of movement elements into a unified and coordinated sequence of actions, which is a fundamental process throughout our lifespan (e.g., typing on our smartphones) [1]. Acquiring new SML requires task repetition, whereby an initial memory trace is established and then consolidated during delayed offline processes without further practice [2,3]. Over the past two decades, numerous approaches have been explored to optimize motor learning, with some suggesting potential benefits following physical or cognitive exercise [4,5].

Recent investigations suggest that physical exercise executed before learning a new motor skill can enhance either its acquisition [6–8], or consolidation [5,9]. While the meta-analysis by Wanner et al. [10] reported limited effects of high-intensity exercise (HIIE) on skill acquisition across various motor paradigms (e.g., visuomotor accuracy tracking, serial reaction time tasks), none of the included studies specifically examined the impact of HIIE on the acquisition of SML. Notably, the widely used finger SML paradigm, which involves the explicit learning of goal-directed finger movements (typically 5–8 items) [3,11] recruits distinct neural substrates during acquisition and consolidation, thereby distinguishing it from motor adaptation paradigms [12,13]. Emerging evidence on the effects of HIIE after acquisition further reinforces this task-specific distinction, suggesting that while consolidation performance improves in visuomotor tasks, SML remains stable [14–16], highlighting task-specific differences in exercise-induced motor learning.

The influence of HIIE on motor learning is often attributed to its associated physiological cascade, including the release of brain-derived neurotrophic factor (BDNF) and lactate accumulation, both of which play a pivotal role in learning-dependent plasticity [17,18]. HIIE has been reported to induce greater releases of both BDNF and lactate compared to low-intensity, or continuous physical exercise [19,20]. This explanation has been supported by Skriver et al. [21], who demonstrated that the magnitude of BDNF and lactate release after HIIE correlates with motor performance during both the acquisition and consolidation of motor skill learning. A similar biological response has been observed after sprint interval exercise (SIE), which effectively induces high lactate levels and BDNF elevation within a shorter exercise duration compared to HIIE. Notably, SIE consists of 12 × 5 s sprints (total exercise time: 1 min) [22], whereas HIIE typically involves 3-min bouts at 80–90% of maximal aerobic power, interspersed with 2-min recovery periods at 25–60% (total: 17 min). SIE offers advantages for the general population as a short, easily accessible exercise, while also providing researchers with a simple, implementable protocol that does not require calibration with a maximal aerobic power test, unlike traditional HIIE. Moreover, SIE has been reported to enhance cognitive performance, with lactate release positively correlating with cognitive improvements [23]. Considering the involvement of cognitive functioning, particularly working memory, in explicit SML acquisition, it is possible that engaging in SIE prior to acquisition may elicit gains in performance.

Cognitive exercise (COG) refers to the practice of a cognitive task involving working memory processes, and has recently been investigated prior to motor learning acquisition, yet to a lesser extent than physical exercise. For instance, Borragán et al. [4] demonstrated that engaging in a COG (i.e., time load dual-back task) before an implicit SML promotes motor skill performance during acquisition. The authors explained that COG may facilitate implicit motor skill acquisition by potentially depleting cognitive resources dependent on the prefrontal cortex, which is less involved in this motor learning acquisition paradigm [4]. More recently, Kimura et Nakano [24] found that COG prior to SML acquisition improved sequence completion time, whereas another study reported no motor performance gains at acquisition [25]. This discrepancy may be due to the greater explicit component in the former study, where COG might have primed prefrontal cortex involvement rather than having induced fatigue. Therefore, determining whether performing COG before explicit SML enhances motor performance during acquisition requires further investigation using a finger SML task that engages prefrontal activation.

Therefore, the present study aimed to investigate whether SIE and COG executed prior to a new explicit SML task may modulate motor performance during the acquisition and consolidation phases (i.e., 24h and 7 days after acquisition). Our primary analyses examined the effects of SIE and COG during the acquisition phase, with the expectation that both groups would outperform the control group. Leveraging the isolated effects of SIE and COG, we further explored their combined effect (SIE + COG) on the acquisition phase. Additionally, motor performance during consolidation was assessed at 24h and 7 days.

## 2. Materials and methods

### 2.1. Participants

Sixty healthy participants (24.9 ± 3.8 years; 31 women) were tested. Seven were left-handed, as assessed by a score < 0.5 on the Edinburgh handedness inventory questionnaire [26]. Inclusion criteria encompassed a Corsi score greater than 4 (6.1 ± 1) (PsyToolkit) [27], and a minimal practice of 2 hours of sport per week, all participants’ information are displayed in Table 1. Individuals with psychiatric or neurological disorders, medical diseases, or current use of medication and/or drug consumption that could potentially affect the cognitive or nervous system were not included. Additionally, individuals with more than five years of musical practice were excluded to minimize potential biases associated with high finger dexterity on SML paradigms. The present research complies with the ethical standards and was approved by the ethical committee of Ile-de-France (10–2023). The recruitment period for this study started on 01/07/2023 and ended on 30/01/2024. All participants signed an informed consent form and were unaware of the study hypotheses. Our study included multiple intervention types, combining COG, SIE, NoExo, and SIE + COG. Given the exploratory nature of this study, we selected a sample of 60 participants, with 15 participants per group. The sample size was determined based on available resources [28].

**Table 1:** Participant characteristics, p-value corresponds to a Kruskal-Wallis test of the factor group.

	NoExo	SIE	COG	SIE + COG	P-value
AGE (years)	24.00 ± 2.59	27.13 ± 4.93	24.00 ± 2.07	24.73 ± 4.33	p = 0.20
HEIGHT (cm)	168.67 ± 10.02	175.00 ± 7.98	174.20 ± 11.33	173.80 ± 9.59	p = 0.21
WEIGHT (Kg)	64.87 ± 8.29	66.20 ± 10.39	67.27 ± 13.69	69.03 ± 12.96	p = 0.85
CORSI	5.71 ± 0.91	6.54 ± 1.20	6.07 ± 1.03	6.08 ± 0.76	p = 0.30
EDINBURGH	0.68 ± 0.65	0.75 ± 0.42	0.83 ± 0.36	0.91 ± 0.23	p = 0.46

### 2.2. The sequential finger tapping task

**Fig 1:**
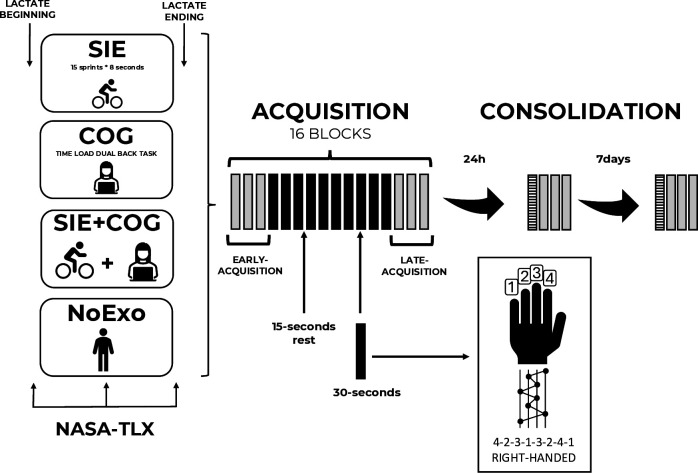
Schematic overview of the experimental design. Participants were assigned to one of the four groups: sprint interval exercise (SIE), cognitive exercise (COG), a combination of sprint and cognitive exercise (SIE + COG) or a No-exercise condition (NoExo). Following groups assignment, there is an illustration of SML blocks of tests and practice during the acquisition and consolidation. Each grey rectangle corresponds to a 30-sec block of the task and represents motor performance assessment at early-and late-acquisition, as well as after 24h and 7 days of consolidation. Black rectangles correspond to the training phase of the task. Hatched blocks correspond to warm-up blocks and were not included in the data analysis. The black hand and the numbers correspond to finger positioning on the keyboard to perform the SML task. All participants performed the task with their non-dominant hand.

The SML was a unimanual finger-tapping task developed on Psytoolkit [29]. Participants were seated 50 cm in front of a 15.6-inch computer screen. The fingers were positioned from left to the right, with the little finger on the number 1 key and the index finger on the number 4 key; this finger configuration was inverted for left-handed participants. The task consisted of performing an explicit eight-movement sequence (4-2-3-1-3-2-4-1) using fingers 2–5 of the non-dominant hand, each finger being associated with a key on the computer keyboard as illustrated in the Fig 1. The sequence was reversed for left-handed participants (1-3-2-4-2-3-1-4). Participants were instructed to repeat the sequence of movements as accurately and as quickly as possible during 30-sec blocks. Each block started with a 3-sec countdown displayed in a white font on a black screen. When the countdown ended, participants started repeating the sequential movement for a period of 30 sec. Each block was followed by 15 sec of rest, during which a white screen was displayed. During each block, we recorded the number of keys pressed, their order, and the timing between each key press using a Psytoolkit script. The dependent variables analyzed were SPEED (i.e., the number of correct sequences per block) and ERROR RATE (i.e., the number of errors per sequence).

### 2.3. Experimental procedure

Participants were randomly assigned to one of the four groups (i.e., 15 participants per group) based on the type of exercise executed before SML acquisition (Fig 1): sprint interval exercise (SIE), cognitive exercise (COG), an alternation of sprint and cognitive exercise (SIE + COG) or a no-exercise (NoExo) condition. Lactate samples were collected from the fingertip using a portable analyser (Lactate Scout, EKF Diagnostics, Cardiff, UK) before and 2 min after each intervention. Moreover, participants completed the National Aeronautics and Space Administration-Task Load Index (NASA-TLX) [30] at the beginning, middle, and end of each intervention to assess the subjective perceptions of mental and physical demands.

Immediately following the intervention, the SML began with an encoding phase during which participants watched a video depicting the sequence order and the correspondence between fingers and the related key to be pressed. Simultaneously, participants were instructed to press the key displayed on the screen to encode the 8-item sequence. Afterward, they were required to perform three correct sequences successively before performing the EARLY-ACQUISITION test, which consisted of three 30-sec blocks. Immediately after, participants repeated the task during an acquisition period of 10 blocks of 30 sec, followed by a LATE-ACQUISITION test of three blocks. EARLY- and LATE-ACQUISITION tests are integral parts of the acquisition phase.

Consolidation was assessed after one day (24h) and a week (7 days) after acquisition. Both tests were performed remotely from participants’ homes in a quiet environment and without distractions [31]. During these consolidation tests, participants performed four blocks of 30 sec (Fig 1). These sessions were guided by video reminders of the task instructions. First, participants completed questionnaires about their bedtime and wake-up times, quality of sleep, and level of sleepiness. Then, they received a short reminder of the task’s goal and completed 4 blocks of practice; each test lasted about 3–4 minutes.

### 2.4. Exercise interventions

#### Sprint interval exercise.

Physical exercise consisted of repeated SIE on a cycle ergometer (Monark, Vansbro, Sweden). Participants warmed up for five minutes on the cycle ergometer, reaching and maintaining a power output between 50 and 75 watts. Final minute of warm-up, participants performed three short accelerations. The workload was then adjusted to the participant’s body weight, with the flywheel resistance being set at 0.07 N·kg^−1^ of body mass, as commonly used for the Wingate test. During sprints, participants were instructed to reach their peak power as quickly as possible and maintain it over the 8-sec sprint duration. Each sprint was immediately followed by 64 sec of rest, using the standardized 1:8 work-to-rest ratio [32]. Participants performed 15 sprints, totaling two minutes of high-intensity exercise within a 16-min intervention period. The NASA-TLX was completed after the 8^th^ sprint.

#### Cognitive exercise.

Participants in the COG group executed the Time Load Dual Back task (TloadDback) [4] They positioned their left index finger on the “W” key and their right index finger between the “L” and “M” keys of a laptop computer with an “AZERTY” keyboard. After a 3-sec countdown, the screen turned black and a succession of digits and letters was alternately displayed every second on the screen. Participants were instructed to press the “L” key when they saw an even digit and the “M” key when they saw an odd digit. When two successive letters (with a digit in between) were similar, participants used their left index finger to press the “W” key. The task lasted for 16 min with a one-minute break in the middle to complete the NASA-TLX.

#### Sprint and cognitive combined exercise (SIE + COG).

Participants followed the same procedures as during the SIE and COG but in an alternating manner. Instead of passive rest periods between each sprint, participants performed the TloadDback task for 60 sec. Immediately after each sprint, the experimenter placed a laptop computer on the handlebars of the ergocycle, and the participant completed the TloadDback task. At the end of the 8^th^ sprint, the TloadDback was not performed to allow sufficient time to complete the NASA-TLX.

#### No exercise (NoExo).

Participants remained seated on a chair approximately 50 cm in front of a screen and watched a 16-min documentary about an animal photographer. After 8 min of watching the documentary, corresponding to the same duration as the other conditions, participants completed the NASA-TLX.

### 2.5 Statistical analysis

All analyses were performed using R software with the {lme4}, {emmeans}, and {r2glmm} packages. The significance level was set at 0.05, and a Tukey post-hoc test was used when a significant factor or interaction was found. Outliers were detected and removed from the model residuals using the first quartile – 1.5 * interquartile range and quartile the third quartile + 1.5 * interquartile range [33]. A participant identified as an outlier in one analysis (e.g., acquisition) was not systematically excluded from other analyses (e.g., consolidation). Since acquisition and consolidation were examined using separate linear mixed-effects models, outlier exclusion was applied independently for each analysis model, based on whether the assumptions of the model (e.g., normality of residuals, homoscedasticity) were satisfied. The linear mixed models (LMM) included random intercepts only, as the inclusion of random slopes led to convergence issues due to the limited sample size. For the four blocks performed at consolidation time (i.e., 24h, 7 days) the first block was considered as a warm-up and was not included in the final analysis (unknown to the participant). For each TEST (EARLY-ACQUISITION, LATE-ACQUISITION, 24h, 7 days), the outcomes of three test blocks were averaged for analysis. Acquisition was analysed using a LMM with the variable ERROR RATE and SPEED, and the fixed effects TEST (EARLY-ACQUISITION, LATE-ACQUISITION) and GROUP (SIE, COG, SIE + COG, NoExo), and subject-specific random effects. Cofactors such as DELTA LACTATE, MENTAL DEMAND, and PHYSICAL DEMAND were included in the initial models. Each model was tested with the cofactors and subsequently re-evaluated by removing them one by one. The validity of the models was assessed using the Akaike Information Criterion (AIC), Bayesian Information Criterion (BIC), and the estimated R². Ultimately, the best-fitting models for each case were the simpler ones without any cofactors. A second LMM was computed to assess consolidation effects with SPEED and ERROR RATE variables, and fixed effect TEST (LATE-ACQUISITION, 24h, 7 days) and GROUP (SIE, COG, SIE + COG, NoExo), and subject-specific random effects. Changes [%] for SPEED and ERROR RATE were calculated for ACQUISITION with the following formula: $\left(\frac{\left(LATE\;ACQUISITION-EARLY\;ACQUSISTION\right)}{EARLY\;ACQUISITION}\right)*100\;$

The changes for 24h and 7 days were also calculated with the same formula using LATE-ACQUISITION as reference timepoint. All the analysis of changes in performance were analyzed using the factor GROUP on a Kruskal-Wallis test. The mental and physical components of the NASA-TLX were analyzed using a DELTA (i.e., END – BEGINNING) as well as lactate (i.e., AFTER EXERCISE – BEFORE EXERCISE) and were analyzed according to the GROUP using a Kruskal-Wallis test as the residuals of linear model did not follow a normal distribution. A significant test for GROUP in Kruskal-Wallis test was more accurately discriminate using a Dunn post-hoc test with Bonferroni method. Finally, some exploratory correlations between DELTA LACTATE and changes for ACQUISITION, 24h and 7days in SPEED and ERROR RATE were attempted using Spearman correlation.

## 3. Results

### SML performance during acquisition

**Fig 2:**
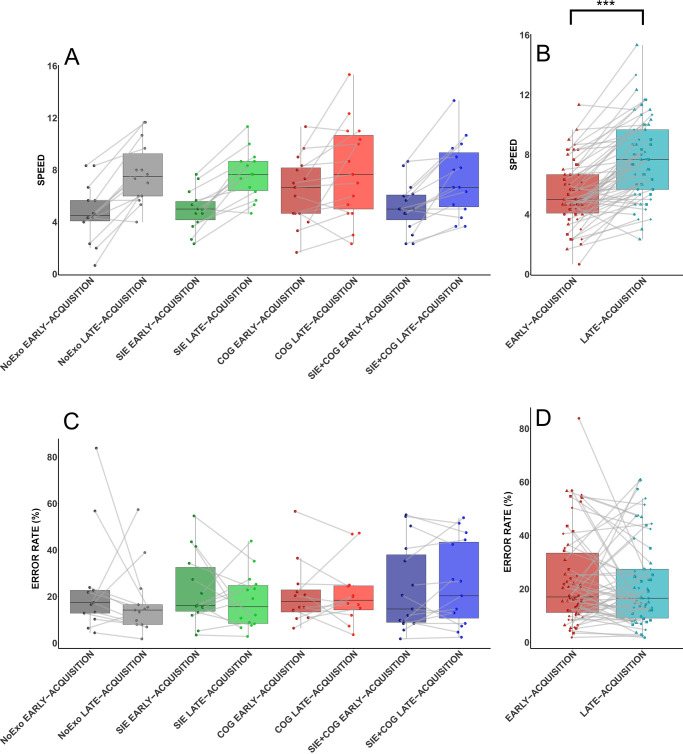
Behavioral performance evolution during acquisition. A. SPEED (i.e., mean number of correct sequences per test) for the EARLY- and LATE-ACQUISITION tests across all groups. In this model, 4 data points out of 120 were identified as outliers and removed from the analysis. B. The SPEED for the EARLY- and LATE-ACQUISITION tests across all groups. C. ERROR RATE during the EARLY- and LATE-ACQUISITION for each group. In this model, 6 data points out of 120 were identified as outliers and removed from the analysis. D. ERROR RATE performed during the EARLY- and LATE-ACQUISITION with all groups. Individual data are represented according to the group: dot for NoExo, triangle for COG, square for SIE, and diamond for SIE + COG. ***: p < 0.001.

No GROUP differences were observed for SPEED (F(3,54) = 1.31; p = 0.28; $\eta_{p}^{2}$ = 0.068) and ERROR RATE during EARLY-ACQUISITION (H(3) = 0.52; p = 0.91; η² = 0.000). For SPEED, the LMM showed a significant main effect of TEST (F(1,48) = 59,91; p < 0.001; $\eta_{p}^{2}$ = 0.555), but no main GROUP effect (F(3,54) = 0.65; p = 0.58; $\eta_{p}^{2}$ = 0.035) or a TEST*GROUP interaction (F(3,48) = 1.14; p = 0.34; $\eta_{p}^{2}$ = 0.067; Fig 2A). Post-hoc analyses revealed an increase in the number of correct sequences from EARLY- to LATE-ACQUISITION (p < 0.001) (Fig 2B). For ERROR RATE, the data analysis did not show any effect of TEST (F(1,52) = 0.09; p = 0.76; $\eta_{p}^{2}$ = 0.002; Fig 2D), GROUP (F(3,58) = 1.15; p = 0.34; $\eta_{p}^{2}$ = 0.056) or a GROUP*TEST interaction (F(3,51) = 0.87; p = 0.46; $\eta_{p}^{2}$ = 0.049; Fig 2C). Finally, no significant differences were observed for changes [%] in SPEED (H(3) = 4.07; p = 0.25; η² = 0.02; SIE = 53%; COG = 30%; NoExo = 58%; SIE + COG = 45%) or ERROR RATE (H(3) = 4.39; p = 0.22; η² = 0.02; SIE = −1%; COG = −20%; NoExo = 4%; SIE + COG = −31%) regarding the GROUP factor.

### SML performance during consolidation

**Fig 3:**
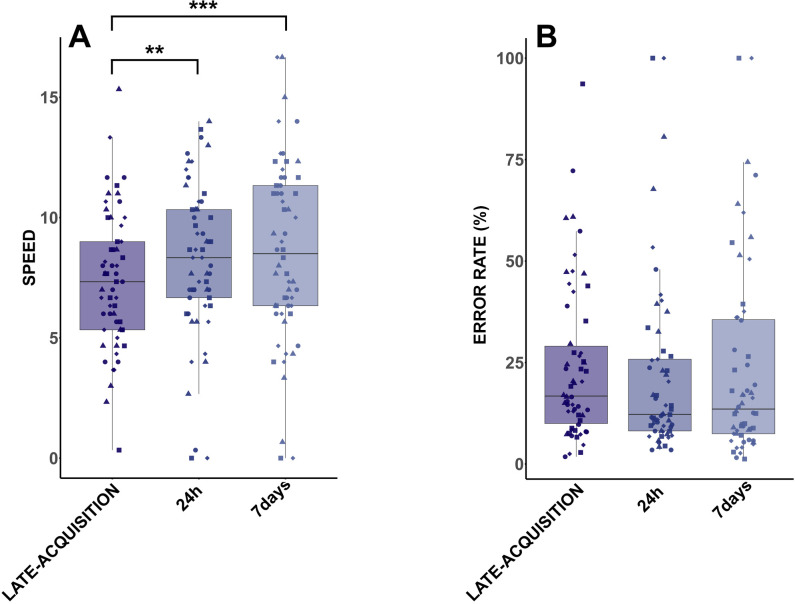
Behavioral performance evolution during consolidation. A. SPEED across the late-acquisition test and consolidation tests with all groups pooled. In this model, 6 data points out of 180 were identified as outliers and removed from the analysis. B. ERROR RATE across the late-acquisition and consolidation tests with all groups pooled. In this model, 7 data points out of 180 were identified as outliers and removed from the analysis. Individual data are represented according to the group dot for NoExo, triangle for COG, square for SIE, and diamond for SIE + COG. **: p < 0.01; ***: p < 0.001.

The LMM showed a significant main effect of TEST (F(2,108) = 10.388; p < 0.001; $\eta_{p}^{2}$ = 0.161), but no main effect of GROUP (F(3,54) = 0.288; p = 0.83; $\eta_{p}^{2}$ = 0.016), and no GROUP*TEST interaction (F(2,108) = 1.058; p = 0.39; $\eta_{p}^{2}$ = 0.055). Tukey post-hoc revealed a significant increase in the number of correct sequences from LATE-ACQUISITION to both 24h (p < 0.01), and 7 days (p < 0.001) tests (Fig 3A). For ERROR RATE, there was no significant effect of TEST (F(2,106) = 1.87; p = 0.16; $\eta_{p}^{2}$ = 0.034; Fig 3B), or GROUP (F(3,54) = 0.8; p = 0.5; $\eta_{p}^{2}$ = 0.042), or GROUP*TEST interaction (F(6,106) = 0.32; p = 0.92; $\eta_{p}^{2}$ = 0.018). The learning curve is presented in the supplementary data (S1 Fig). Changes in SPEED were not different between GROUP for 24h (H(3) = 0.59; p = 0.89; η² = 0.04; SIE = 9%; COG = 17%; NoExo = 13%; SIE + COG = 6%) as well as for 7 days (H(3) = 1.94; p = 0.59; η² = 0.02; SIE = 11%; COG = 14%; NoExo = 31%; SIE + COG = 9%). Finally, changes in ERROR RATE were equivalent for all groups at 24h (H(3) = 1.03; p = 0.79; η² = 0.03; SIE = − 9%; COG = 1%; NoExo = 0%; SIE + COG = − 11%), and at 7 days (H(3) = 0.67; p = 0.88; η² = 0.04; SIE = 1%; COG = − 25%; NoExo = − 15%; SIE + COG = − 23%) tests.

#### Psychophysiological effect of interventions.

**Fig 4:**
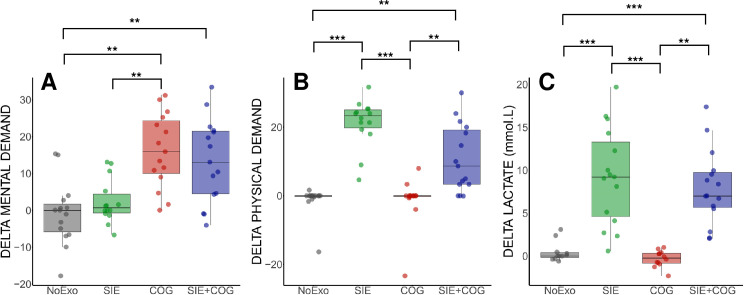
Changes in MENTAL DEMAND (A), PHYSICAL DEMAND (B), and LACTATE (C). Δ values were calculated as the difference between END and BEGINNING assessments for each exercise, evaluated using NASA-TLX. (A) MENTAL DEMAND showed significant changes in SIE + COG and COG groups compared to SIE and NoExo. (B) PHYSICAL DEMAND significantly increased in SIE and SIE + COG compared to NoExo and COG. (C) LACTATE levels significantly changed in SIE and SIE + COG compared to COG and NoExo. Significant differences: ** p < 0.01; *** p < 0.001.

Data from the NASA-TLX showed a significant effect of GROUP for MENTAL DEMAND (H(3) = 22.47; p = 0.001; η² = 0.348). Post-hoc analysis revealed that MENTAL DEMAND was significantly higher in the COG group compared to SIE and NoExo (p < 0.01), while SIE + COG also showed higher MENTAL DEMAND than NoExo (p < 0.01) but not SIE (p = 0.09), with no significant difference between SIE + COG and COG (p = 1; Fig 4A). A similar pattern was observed for the PHYSICAL DEMAND which showed a significant GROUP effect (H(3) = 39.25; p = 0.001; η² = 0.647), with higher values in the SIE group compared to NoExo or COG groups (p < 0.001), and in SIE + COG compared to NoExo and COG (p < 0.01), while no significant difference was found between SIE and SIE + COG (p = 0.29) and between COG and NoExo (p = 1; Fig 4B). Finally, DELTA LACTATE analysis yielded a significant difference between GROUP (H(3) = 36.33; p < 0.001; η² = 0.595). Post-hoc showed higher LACTATE changes for SIE compared to COG and NoExo (p < 0.001), as well as SIE + COG compared to COG and NoExo (respectively p < 0.01 and p < 0.001), while no difference was found between COG and NoExo (p = 1), and SIE and SIE + COG (p = 1; Fig 4C).

Finally, no correlations were found between DELTA LACTATE and changes [%] in ERROR RATE or SPEED. For SPEED, correlations were not significant during ACQUISITION (r(51) = 0.1; p = 0.41), from LATE-ACQUISITION to 24h (r(50) = −0.02; p = 0.87), and from LATE-ACQUISITION to 7 days (r(51) = 0.04; p = 0.75). Similarly, for ERROR RATE, no significant correlations were observed during ACQUISTION (r(48) = −0.06; p = 0.66), from LATE-ACQUISITION to 24h (r(49) = 0.03; p = 0.83), and from LATE-ACQUISITION to 7 days (r(51) = 0.17; p = 0.22).

## 4. Discussion

The present study examined the effects of SIE, COG, and alternating SIE + COG on motor performance during acquisition and consolidation of explicit SML. The main findings indicated that all experimental groups showed similar improvements in the number of correct sequences performed following SML acquisition, which further enhanced during the 24h consolidation period and stabilized over one week (7 days), while the error rate remained stable. These findings suggest that neither physical, cognitive, nor combined exercise prior to explicit SML provided further benefits on motor performance during acquisition or subsequent consolidation processes when compared to a control intervention.

Our results showed that SIE performed before SML did not lead to additional performance gains during acquisition, or consolidation compared to the NoExo group. Although our findings contrast with the few studies using visuomotor tracking paradigms, where physical exercise before learning has been shown to enhance performance at either the acquisition [6,7] or consolidation stage [5]. Our results align more closely with recent studies on explicit SML paradigms, which have shown that HIIE performed after acquisition has no substantial impact on consolidation [14–16]. Therefore, our study reinforces the current reports suggesting that explicit SML might not benefit from high-intensity exercise (HIIE or SIE), whether performed before or after acquisition. Aside from differences in the motor paradigms, the primary discrepancy lies in the type of physical exercise, as our study used the shortest protocol with SIE (2 min cumulative), whereas other studies reporting SML benefits used longer exercise protocols such as moderate aerobic exercise (30 min) [7], HIIE (15 min) [8], 20 min [21] or continuous high intensity (15 min) [34]. Therefore, the brief duration of our physical exercise may have been insufficient to influence motor performance during acquisition. While we did not measure BDNF release in the present study, we observed a typical increase in lactate levels following SIE [22,23], and one may speculate that the duration of BDNF release and sustained high blood concentration may have been insufficient to elicit the expected behavioral changes. This hypothesis is supported by a meta-analysis highlighting greater peripheral BDNF elevations following longer durations of physical exercise (e.g., 30 min) [35]. In our study, lactate levels did not significantly correlate with behavioral performance during acquisition or consolidation, whereas Skriver et al. [21] reported a link between lactate elevation and learning during acquisition. However, this finding has yet to be replicated, and a recent review highlighted the complexity of establishing a clear relationship between lactate elevation in motor learning enhancement [36]. Although our main focus was on the impact of SIE on acquisition and exploration of consolidation, the timing of physical exercise is important; when performed before learning, it could enhance both acquisition and consolidation, whereas if performed after acquisition, it might only influence consolidation [37]. To date, convergent findings indicate that physical exercise promotes motor performance during consolidation as the skill becomes more automated and requires fewer cognitive resources [5,10].

Our finding that COG did not yield performance benefits during acquisition and consolidation of SML compared to NoExo contrasts with findings of Borragán et al. [4] and Kimura & Nakano [24]. Discrepancies in findings between studies may stem from differences in the nature of SML acquisition, which was encoded implicitly in Borragán et al. [4] and Kimura & Nakano [24] and explicitly in our study. The distinction between implicit and explicit SML is crucial to consider as the former primarily involves striatal activity, whereas the latter requires more cognitive resources from the prefrontal cortex to learn the sequence of movements [38–40]. Accordingly, Borragán et al. [4] suggested that depleting cognitive resources in the prefrontal cortex by means of COG could alleviate competition between explicit and implicit components of SML, which facilitates striatal involvement, and promotes implicit SML acquisition. In our study, we hypothesize that rather than producing a beneficial priming effect, COG exercise may have taxed cognitive resources needed for encoding explicit SML or may not have been complex enough to influence motor performance during acquisition and, in turn consolidation. Unlike Borragán et al. [4], where the COG difficulty was adjusted based on the participant’s performance by modulating the time of stimulus presentation, the difficulty level of the TloadDback was identical across all subjects in our study. Taken together, we believe that both the nature of the SML and/or the complexity of the COG task may influence motor performance during SML acquisition. Based on the limited studies in this line of research, it seems more plausible that COG enhances motor performance during implicit SML acquisition, but not in explicit SML.

Finally, the alternation of SIE and COG did not influence motor performance during acquisition and consolidation of explicit SML. This result is consistent with other observations showing that neither SIE nor COG alone influenced on explicit SML. Interestingly, in constrast to an acute SIE + COG intervention, Heisz et al. [41] reported that six weeks of daily 20 min sessions of high-intensity interval physical exercise combined with 20 min of COG did not significantly improve performance on a declarative memory task compared with physical exercise alone. However, both physical and COG exercises alone, as well as physical exercise combined with COG, demonstrated significantly better performance compared to a control group.

As with any research, this study has limitations that should be considered when interpreting the findings. First, the absence of significant differences between groups should not be interpreted as evidence for the absence of an effect. Rather, it reflects a lack of sufficient evidence to reject the null hypothesis. We acknowledge that one key limitation of this study is the relatively small sample size, which results in limited statistical power to draw definitive conclusions. Therefore, we emphasize the exploratory nature of this work. Another limitation is the inability to monitor participants’ physical activity after the experiment. While they were instructed to limit exercise for 24 hours, subsequent resumption of normal activity afterward may have influenced consolidation outcomes. However, this likely reflects real-life conditions, particularly in a young, active population. An additional limitation is the exploratory nature of our consolidation performance analyses, which were conducted remotely. Although remote testing may introduce potential distractions, the short duration of the test likely minimized this risk, and its reliability has been validated in previous studies [42,43]. Finally, we acknowledge a limitation in the assessment of participants’ fitness, which was based solely on self-reported weekly physical activity hours. Considering recent studies suggesting a link between fitness level and the effects of exercise on motor learning [15], a more accurate assessment using validated tools such as the GPAQ, IPAQ, or an incremental test would have been more appropriate.

In conclusion, this study examined the effects of SIE, COG, and their combination on SML acquisition and consolidation, yet the results do not support the notion that pre-SML exercise, regardless of type, enhance motor performance in either phase. From a practical perspective, this study contributes to clarifying the understanding of the efficacy and limitations of non-invasive and easily implementable approaches, such as SIE or cognitive training, on explicit motor skill learning.

## Supporting information

S1 File Supplementary data can be found online at: https://osf.io/caqyj/?view_only=2934b161992e462b98b5e489512dbc01
(DOCX)
