# Supplementary material for: The effects of acute physical and cognitive exercises on sequential motor skill learning: An exploratory study
Source: PLoS One. 2025 Jul 11;20(7):e0327725. doi: 10.1371/journal.pone.0327725 (PMC12250563; doi:10.1371/journal.pone.0327725)
Supplement: S1 File — Supplementary data can be found online at: https://osf.io/caqyj/?view_only=2934b161992e462b98b5e489512dbc01 (DOCX) [file pone.0327725.s001.docx]

**SUPPLEMENTARY MATERIALS**

***DATA ANALYSIS***

*Complementary analysis on motor performance*


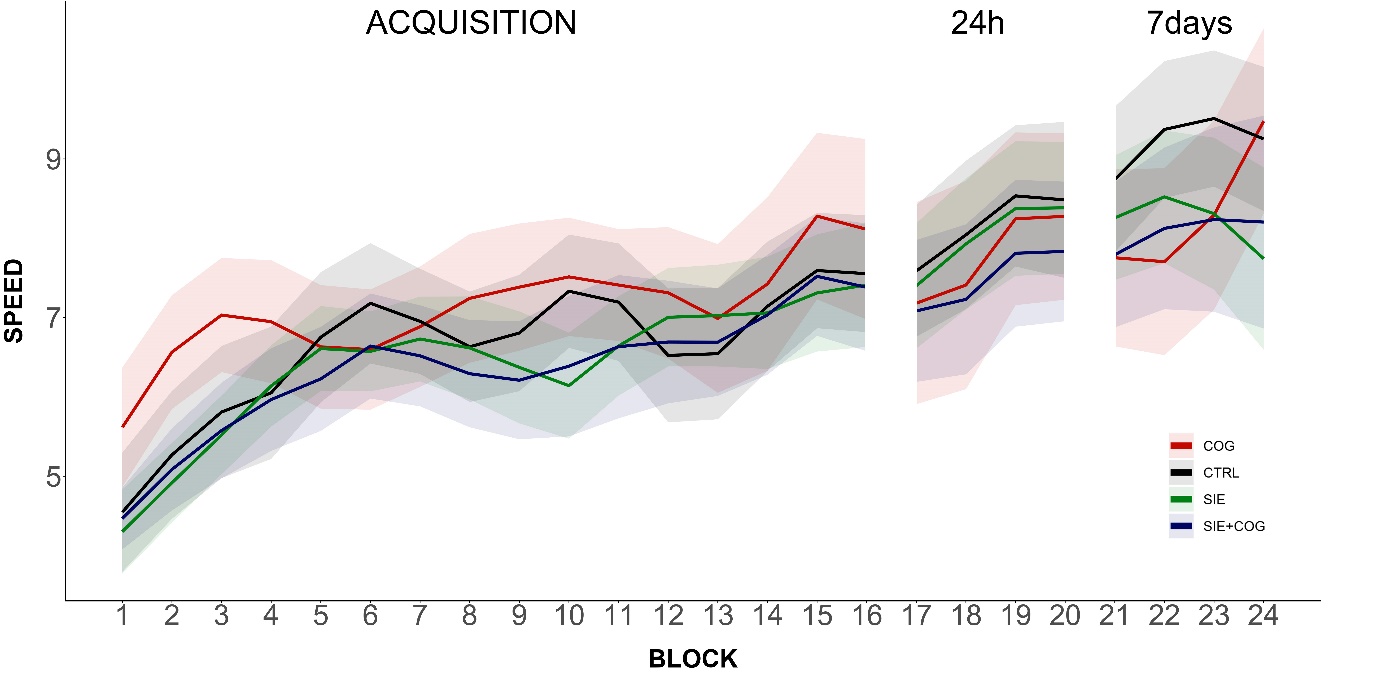


**Figure S1 |** represents the curve of performance for SPEED (i.e. number of correct sequence). Green curve represents SIE, Black curve represents NoExo group, red curve represents COG while the blue curve represents SIE+COG. Shadow represents the SEM.
